# An Experimental Model for the Study of Underwater Pressure Waves on the Central Nervous System in Rodents: A Feasibility Study

**DOI:** 10.1007/s10439-021-02898-6

**Published:** 2021-12-14

**Authors:** Mattias Günther, Ulf Arborelius, Mårten Risling, Jenny Gustavsson, Anders Sondén

**Affiliations:** 1 Department of Clinical Science and Education, Karolinska Institutet, Stockholm, Sweden; 2 Experimental Traumatology Unit, Department of Neuroscience, Karolinska Institutet, Biomedicum – 8B, 171 77 Stockholm, Sweden

**Keywords:** Underwater blast injury, Sprague–Dawley, Experimental model, Electroencephalography

## Abstract

Underwater blast differs from blast in air. The increased density and viscosity of water relative to air cause injuries to occur almost exclusively as primary blast, and may cause disorientation in a diver, which may lead to inability to protect the airway and cause drowning. However, cognitive impairments from under water blast wave exposure have not been properly investigated, and no experimental model has been described. We established an experimental model (water shock tube) for simulating the effects of underwater blast pressure waves in rodents, and to investigate neurology in relation to organ injury. The model produced standardized pressure waves (duration of the primary peak 3.5 ms, duration of the entire complex waveform including all subsequent reflections 325 ms, mean impulse 141–281 kPa-ms, mean peak pressure 91–194 kPa). 31 rats were randomized to control (*n* = 6), exposure 90 kPa (*n* = 8), 152 kPa (*n* = 8), and 194 kPa (*n* = 9). There was a linear trend between the drop height of the water shock tube and electroencephalography (EEG) changes (*p *= 0.014), while no differences in oxygen saturation, heart rate, S100b or macroscopic bleedings were detected. Microscopic bleedings were detected in lung, intestines, and meninges. Underwater pressure waves caused changes in EEG, at pressures when mild hemorrhage occurred in organs, suggesting an impact on brain functions. The consistent injury profile enabled for the addition of future experimental interventions.

## Introduction

Underwater blast injuries were first described in 1917. During WWII, thousands of soldiers and civilians were injured or killed by underwater blast, which led to the development of safety standards to prevent injury or death. Most safety standards had little or no scientific basis. A US Navy 500 psi (3447 kPa) guideline, which was intended to provide a peak pressure at which injuries were likely to occur, was based on unconfirmed assertions that later propagated throughout the literature.^12,13^ While no universal standard for underwater blast safety currently exists, the guideline developed by Richmond *et al*. seems to be the most commonly applied today.^14^ Underwater blast differs from blast in air. Explosions in air typically injure through any of four general categories: primary blast from direct effects of the blast wave; secondary blast from energized projectiles, tertiary blast from whole body translation and quaternary blast from effects of inhaled gases and other sources.^5^ The increased density and viscosity of water relative to air cause underwater blast injuries to occur almost exclusively as primary blast. This type of injury is the result of the energy of the blast wave interacting with the tissues of the human body.^12^ Similar to air blast, the gas-containing organs are the most affected in underwater blast.^13^ There is limited experience on how underwater blast waves influence the brain. Underwater blast may cause disorientation and balance impairments to a diver, which could lead to inability to protect the airway and cause drowning. While animal studies have been performed since 1944 to assess the spectrum of injury, summarized in a comprehensive review,^12^ cognitive impairments from underwater pressure wave exposure have not been properly investigated, and no experimental models for this purpose have been described. The aim of this study was to describe an experimental model for underwater pressure wave exposure to rodents, and to investigate neurological manifestations in relation to demonstrated organ damages. We hypothesized that the model may be useful for studies of graded effects of isolated blast waves.

## Material and Methods

The study was conducted in accordance with the Swedish regional Ethics Approval Board for Animal Research (approval number A89-03). 31 female Sprague-Dawley rats weighing a mean 207 (range 193–218) grams were randomized to control (*n* = 6), exposure 90 kPa (*n* = 8), 152 kPa (*n* = 8), and 194 kPa (*n* = 9).

### The Pressure Wave Generator

**Figure 1:**
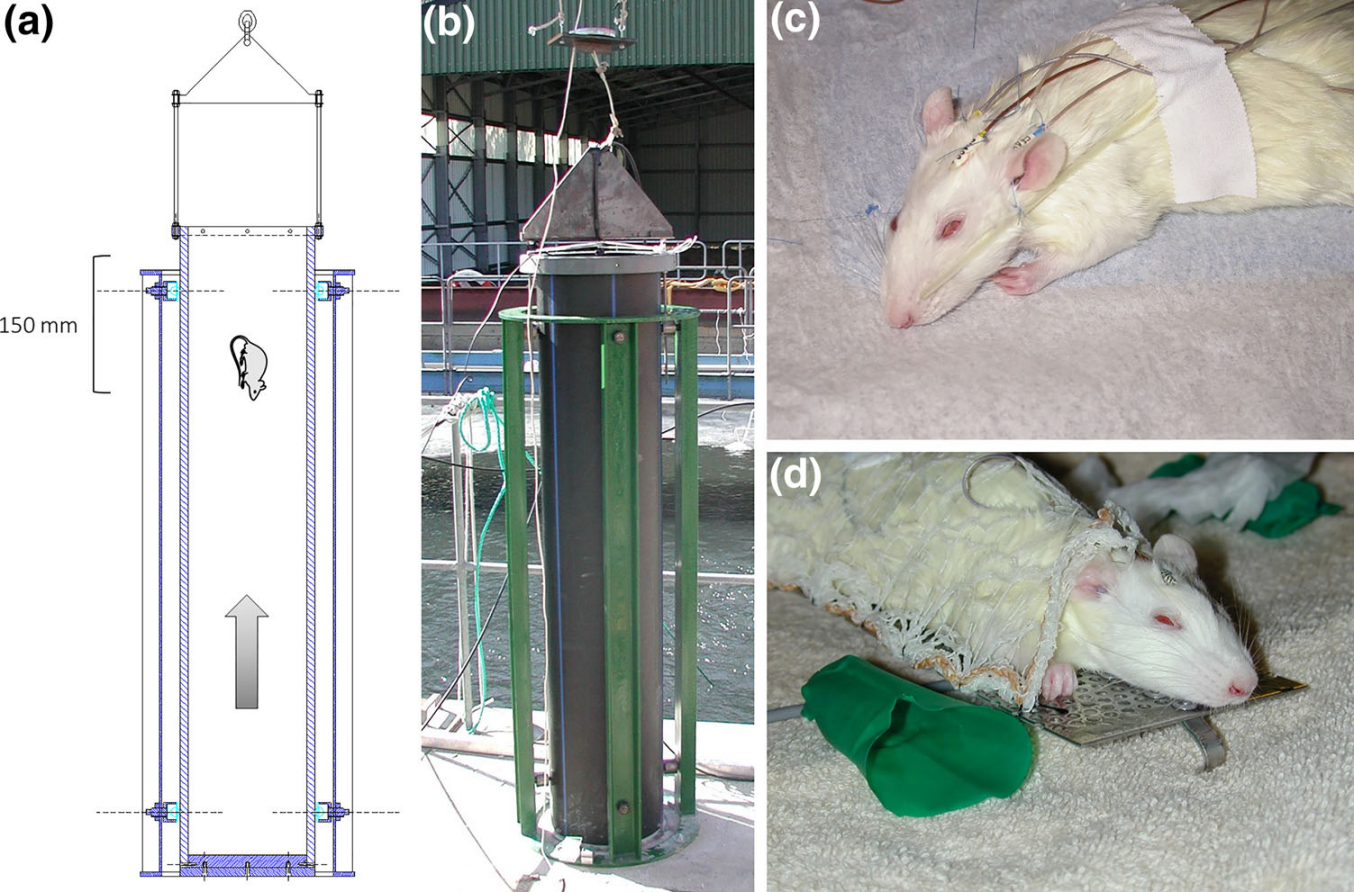
(a) Construction drawing of the water shock tube. The rats were placed 150 mm below the water surface, nose down. The water shock tube was dropped on a granite slab from 10-30 mm height. The impact generated a pressure wave which was propagated through the water (arrow). (b) photo of the water shock tube. (c) The rats had an EEG attached to the skull and oxygen was delivered by a tube fixed to the nose. (d) The rats were fixed on a holder and the airways were secured during submersion by a mask made of a rubber-glove.

The pressure wave generator, (water shock tube) consisted of a polyethylene tube (PE100, inner diameter 280 mm, thickness 18 mm, length 1500 mm) with an aluminum base (thickness 50 mm) (Figs. 1a and 1b). The tube was filled with water to a height of 1.45 meter. The tube was hung from the ceiling with a turnbuckle, to elevate the tube and an electromagnet which was controlled by a power unit, to allow a controlled release. Under the vertical tube, a flat-cut granite slab was placed and fixed into a slightly larger hole, which was drilled in the concrete floor. The tube was lifted to a predefined height (maximum 100 mm) and released by means of the electromagnet. When the water shock tube hit the granite slab, a pressure wave was generated at the bottom, which propagated through the water in the tube. To ensure a clean parallel plane hit between the bottom of the tube and the granite, guide wheels were mounted on the frame. The maximum pressure of the pressure pulse generated in the device was controlled by the drop height. The duration of the pulse was quantified from the measured waveform. Mathematical calculations of the water shock tube were described in a Swedish Defense Research Agency report.^2^ Measurements of the pressure profile (150 mm below the water surface) were performed for 10-, 20- and 30-mm drop heights. Repeated measurements at 10-, 20- and 30-mm drop heights were performed to calculate the average of the maximum pressure and the areal impulse (integral of the pressure wave and time) of the initial pulse. The shock gauge transducer was a TR-11 (Neptune Sonar, East Yorkshire, Great Britain). The hydrophone was based on a piezoelectric tourmaline crystal connected to a miniature low noise coaxial cable, with a dynamic measurement pressure range 0–275 MPa and rise time < 4 *μ*s. The sampling frequency was 1 MHz, for accurate determination of the shock wave time-pressure profile.^16^

### Anesthesia and Preparation

The rats were anesthetized with an injection into the abdominal cavity of a mixture of Hypnorm (fentanyl 0.315 mg/mL; fluanisone, 10 mg/mL), midazolam (5 mg/mL) and water (1 + 1 + 2), dose 0.24 mL per 100 grams body weight. To maintain anesthesia, fentanyl/fluanisone 0.02 mL per 100 grams of body weight was administered every 30 min. To prevent from hypothermia, the rats were placed on a heating pad controlled by a temperature sensor in the rectum, after anesthesia and during all parts of the experiment when possible (CMA/150 Temperature Controller CMA Microdialysis AB, Sweden). Electroencephalography (EEG) electrodes were applied to the scalp: in the midline, over the frontal lobe, over the occipital lobe and over each temporal lobe (Fig. 1c). Two additional electrodes were applied as reference electrodes in each shoulder. All electrodes were sutured to the skin. The EEG was recorded with four channels (EEG-7209, Nihon Kohden Corporation, Japan). Blood oxygen saturation and animal heart rates were recorded using a pulse oximeter attached to the sole of one hind leg with tape (8600V Series Veterinary Pulse Oximeter, Nonin Medical Inc, USA). An oxygen tube was attached with a single suture so that it allowed oxygen flow at the nose. Oxygen flow was set to 0.5 L per minute. The rats were placed on a fixture and secured with a net. The airway was secured by placing a hood, made from a thumb of a Biogel glove over the head, without covering the ears (Fig. 1d). The hood was tied together in front of the nose. An overpressure in the open mask allowed for outflow of oxygen at the back of the mask, while limiting the air inside of the mask as much as possible to prevent from pressure wave distortion. Randomization to groups was then performed by sealed envelope.

### Pressure Wave Exposure

The water shock tube was filled with room temperate water to 50 mm from the upper edge. The tube was raised by means of a turnbuckle attached to an electromagnet 10, 20 or 30 mm above the foundation. The rats were immersed in the vertical tube and fixed so that the nose was 150 mm below the water surface. The water shock tube was released after 30 s. To avoid motion artifacts on the EEG in direct connection with the exposure, the rats remained under water for an additional 60 s. The rats were then lifted, and the mask removed. If water was detected in the airways, a sign of incomplete hood closure, animals were excluded from further analysis (*n* = 7). Five minutes after exposure, the rats were removed from the fixture and transferred to the heating pad. EEG recording, respiration, heart rate and blood oxygenation continued for 30 min, continuously for the first 10 min then every 5 min. Control animals were treated identically, except for that the tube was never released.

At completion of the experiments, the animals were sacrificed with a double anesthetic dose of fentanyl/fluanisone-midazolam. The abdomen was opened, the inferior vena cava was cut open and the blood was stored for analysis of calcium-binding protein B (S-100b). Brain, lungs, and intestines were examined for signs of visible bleeding. The brain, right lung, and appendix (caecum) were fixed in formalin (4%) and sucrose (10%), for the first 5 animals per exposed group and 4 controls, for sectioning for histopathological examination with light microscopy. The tissue was sectioned in 14 µm thick sections, the brains in coronal orientation, the complete caecum, and the middle part of both lungs. The slides were blinded to an assessor for quantification. EEG was analyzed by a board-certified, clinically active, senior clinical specialist in neurophysiology. The EEG was blinded to the assessor.

### Statistical Analysis

Statistical analyses were done using GraphPad Prism version 9.1.0 (GraphPad Software). The primary outcome was number of exposed animals with EEG changes compared. p<0.05 was considered significant. Plotted bars display the standard deviation. Number of animals with changes in EEG, lung, intestines, and meninges were analyzed with chi-square test for trend (Cochran-Armitage method). SpO_2_, heart rate, and temporal EEG were analyzed with mixed-effects model (REML). S100b was analyzed with ANOVA and Tukey´s multiple comparisons test. Correlation between peak pressure and impulse was done with linear regression.

## Results

### The Water Shock Tube

**Figure 2:**
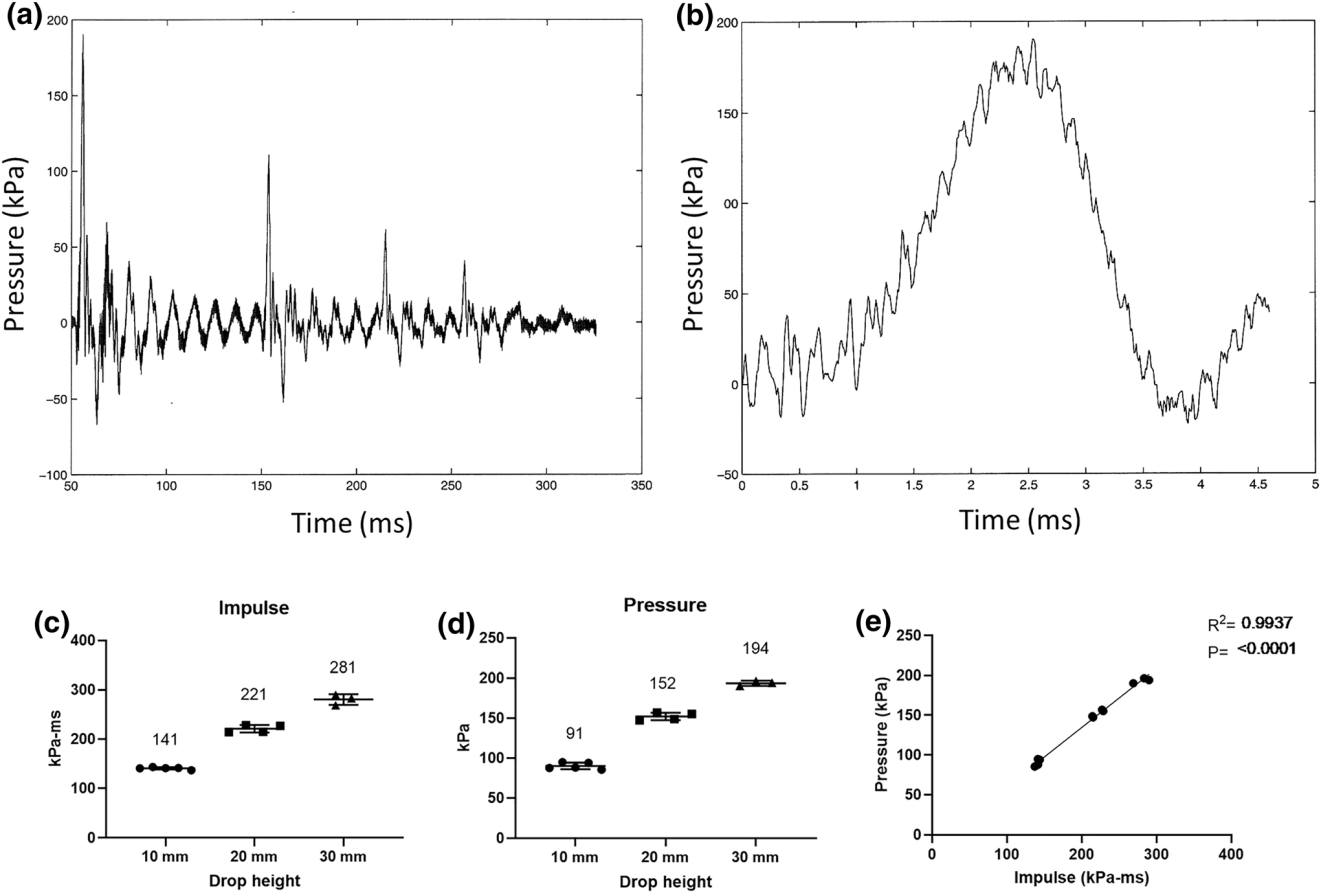
Representative pressure waves generated by 30 mm drop height, displayed for 300 ms in (a), and for the initial 5 ms in (b). The pressure sensor was placed 150 mm below the water surface. (c) the areal impulse a was mean 141 kPa-ms for 10 mm, 221 for 20 mm and 281 for 30 mm drop height. (d) The pressure was a mean 91 kPa for 10 mm, 152 kPa for 20 mm and 194 kPa for 30 mm drop height. (e) A linear regression of peak pressure and impulse showed a strong correlation (*R*^2^=0.99, *p* ≤ 0.0001).

The water shock tube produced a pressure wave, when hitting ground, with a duration of the primary peak of 3.5 ms, and the duration of the entire complex waveform including all subsequent reflections of 325 ms. Representative pressure waveforms are shown for the 30 mm drop height (Figs. 2a and 2b). Three drop heights were chosen to produce appropriate blast waves: 10, 20 and 30 mm. The mean areal impulses produced were (with increasing drop height): 140.8 (SD 2.4), 221.2 (SD 7.7) kPa-ms and 280.7 (SD 10.1) kPa-ms (Fig. 2c). The corresponding means of maximum pressures recorded were: 90.1 (SD 4.2) kPa, 152.1 (SD 4.7) kPa and 193.7 (SD 3.2) kPa (Fig. 2d). A linear regression of peak pressure and impulse showed a strong correlation (*R*^2^ = 0.99, *p* ≤ 0.0001) (Fig. 2e).

### Cardiorespiratory System

**Figure 3:**
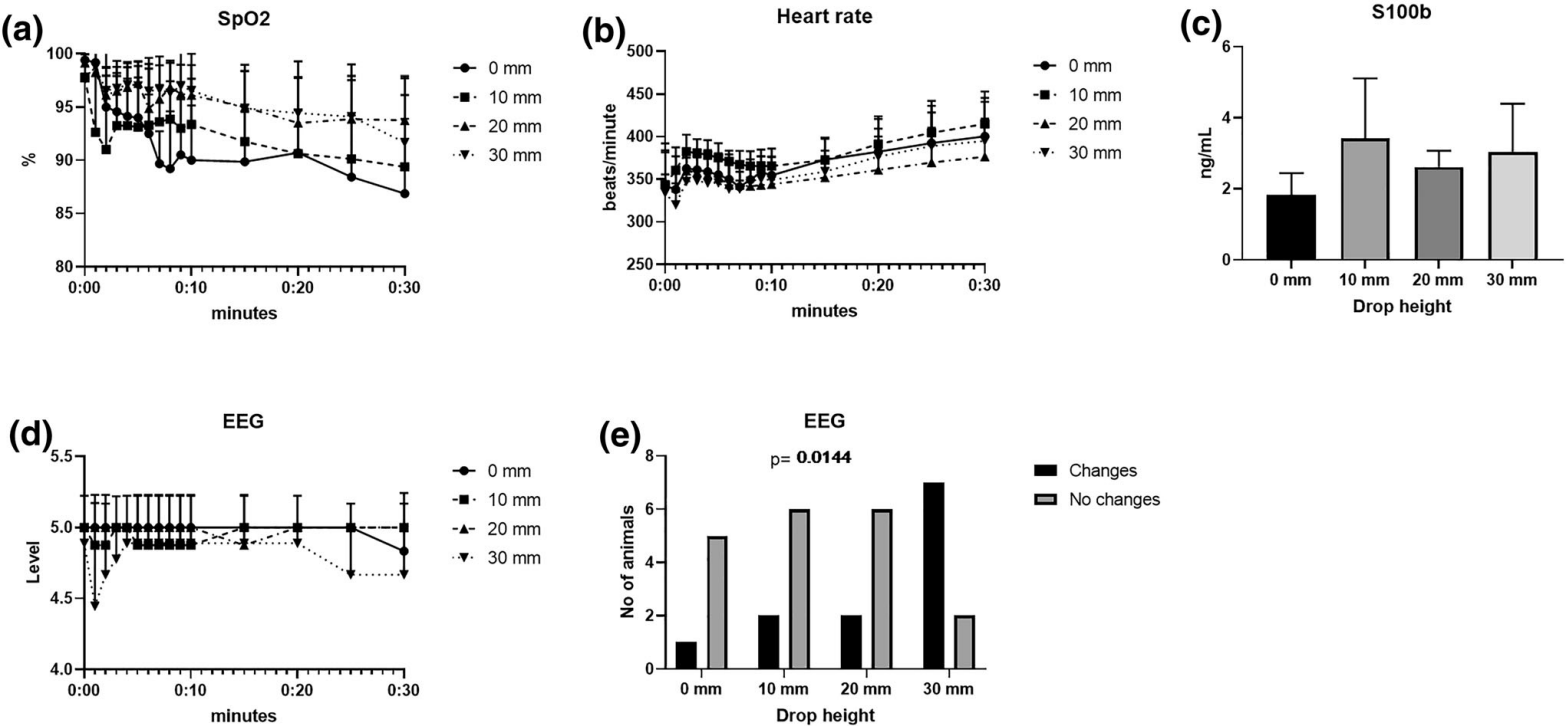
(a) SpO_2_ was > 87% during the 30 min observation period, with no significant difference between groups, (b) Heart rates did not differ between groups, (c) S100b did not differ between groups, (d) EEG displayed changes, mainly during the first 2 min following the pressure wave. (e) a dose-response was displayed in EEG changes, with increasing number of animals affected correlating to drop height (*p* = 0.014).

**Figure 4:**
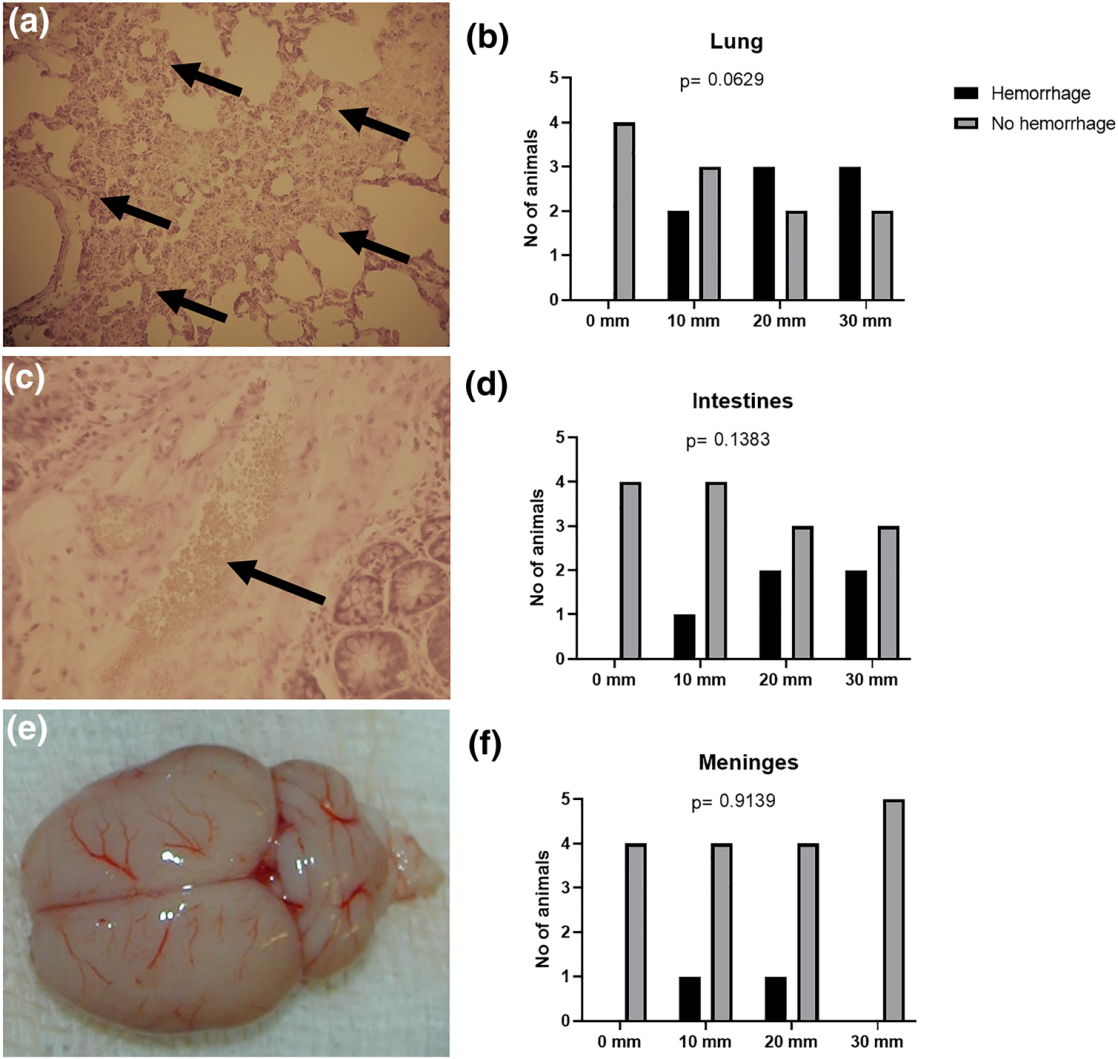
(a) Photomicrograph of lung tissue, showing microscopic hemorrhage (arrows). (b) No difference was found between groups. (c) Photomicrograph of intestines, showing microscopic hemorrhage (arrow). (d) No difference was found between groups. (e) Photograph of the brain, showing macroscopic hemorrhage. (f) No difference was found between groups. X-axes are drop heights

Oxygen saturation (SpO_2_) varied between 87% and 100% during the experiments, and no differences were seen between groups (Fig. 3a). No macroscopic damage to the lungs or macroscopic bleedings were detected (data not shown). There were no significant differences in microscopic bleedings in the lungs (*p* = 0.063) (Figs. 4a and 4b). Heart rates varied between 325 and 400 beats per minute, and no differences were seen between groups (Fig. 3b).

### Central Nervous System

There were no significant differences in S100b in blood (*p* = 0.253) (Fig. 3c). No signs of bleeding in the brain were demonstrated either macroscopically or by microscopy. No significant differences were observed in hemorrhage of the meninges (*p* = 0.914) (Figs. 4e and 4f).

### EEG Analysis

The EEG recordings were analyzed for patterns classified according to five different easily defined levels of activity. The pre-exposure EEG pattern (baseline pattern) was similar in all animals and was dominated by irregular 4 to 7 Hz activity, with an amplitude maximum observed in the post-central hemispheric quadrants of about 100 µm. Fast activity in the β-range frequency of low amplitude was detected bilaterally over the temporal regions. The baseline pattern was observed throughout the observation period in control animals. Animals subjected to underwater blast displayed within 90 s a general slowing in frequency range of the EEG band to a dominating 2–5 Hz activity (slow frequency range pattern) or a more marked slowing in frequency range of the EEG band to a general 1–3 Hz activity (marked slow frequency range pattern). These EEG changes were in most cases transient within minutes but continued in one case and occurred again in three cases during the 30 min observation period. In five of the exposed animals the EEG changes were observed after the animals had been taken out of the water tank. The EEG patterns were quantified on an ordinal scale (levels 0-5, with level 5 being baseline pattern, and level 0 being marked slow frequency range pattern) to visualize the duration of the changes (Fig. 3d). The number of animals with a difference in EEG displayed a linear trend between the drop height (0 mm, 10 mm, 20 mm, 30 mm and the fraction of animals (*p* = 0.014) (Fig. 3e).

### Intestines

No macroscopic bleeding was found in the gastrointestinal tract. There were no significant differences in microscopic bleedings in the intestines (*p* = 0.138) (Figs. 4c and 4d).

## Discussion

In this study we describe an experimental model for investigating the effects of underwater pressure waves on rodents. Using the model, we demonstrate that underwater pressure waves cause EEG changes at pressure levels by which microscopic hemorrhage in organs occurred.

The consequences of a pressure wave are determined by the peak positive pressure, duration of the overpressure, total energy of the blast, and areal impulse (the maximum area under the pressure-time curve).^12^ For ideal air blasts, the Friedlander waveform allows for a comprehensive description of blast intensity through two parameters, peak pressure, and positive phase duration. Many currently available air blast injury criteria use these two parameters to describe exposure and therefore risk of injury.^4^ Underwater blast waves are more complex, and involve bottom reflections, charge depth, bottom depth, gage depth, bottom reflectivity, and gas bubble fluctuations (cavitation) following detonation.^13^ Therefore, standard Friedlander equations cannot be used for the complex waveforms from underwater blasts because the entire shape of the curve is much more variable and a positive phase duration may be difficult to determine.^8,13^ The difficulty in precisely calculating or predicting impulse has led to guidelines based on range or peak pressure, even though these factors may be insufficient.^13^ In our experimental model, peak pressure and impulse correlated well, and either one could be used to predict the injuries, which contrasts the assertions of early reports stating that biological effects may be better correlated to the pressure wave's impulse than maximum pressure.^1^ Impulse has become the standard for predicting destruction of buildings and other structures, but little experimental data has supported the same assertion for physiological damage.^12^ Both peak pressure and impulse may be necessary to accurately predict injury risk, similar to the use of both peak pressure and overpressure duration to predict injury risk in air blast.^13^

For this study, we selected peak pressures based on our earlier studies of air pressure wave effects on swine brains. Air pressure waves of 237 kPa gave pronounced effects on EEG for 5–15 s, and changes for up to 2 min.^3^ The highest pressure in the water shock tube, 194 kPa, was within the pressure range where effects on the brain were estimated to occur. The first peak pressure had a duration of 3.5 ms, which may be slower than in air blast using explosives, where peak pressures may be in the range of 0.5 ms, although longer peak pressures are also possible.^7^

Investigating EEG in animals under general anesthesia requires some considerations. General anesthesia produces distinct patterns on the EEG, the most common of which is a progressive increase in low-frequency, high-amplitude activity as the level of general anesthesia deepens.^6^ We deemed anesthesia by fentanyl/fluanisone-midazolam to be the most appropriate for accurate EEG analyzations. Fentanyl/fluanisone-midazolam is well tolerated by rats and has low confounding effects on somatosensory evoked potentials, an electrophysiological technique that provides an objective and non-invasive measure of neurological function.^10^ Unconsciousness induced by fentanyl/fluanisone-midazolam is associated with slower EEG patterns, while the NMDA antagonist ketamine is associated with active EEG patterns.^6^ Fentanyl/fluanisone has stimulating effects on the amount of spike-wave discharges in rats, but not in a dose-dependent manner.^11^ Midazolam, which enhances the effect of the neurotransmitter GABA on the GABA_A_ receptors, may increase beta activity on the EEG (13 to 25 Hz).^6^ The baseline pattern after induction of anesthesia was similar in all animals and dominated by irregular 4 to 7 Hz activity. Humans have a normal, active EEG, with prominent alpha activity (10 Hz) before induction of anesthesia, with eyes closed.^6^ The EEG changes we observed after exposure to underwater blast waves differed distinctly from the baseline pattern and were interpreted as activity equivalent to spatial disorientation but not unconsciousness, in unanesthetized animals. However, the general anesthesia prevented from evaluations of more specific effects on the brain. It would be technically challenging to submerge unanesthetized rodents. Therefore, future studies may include more advanced neuro-monitoring such as cerebral microdialysis. It is also possible to include behavioral studies, and the experimental model allows for survival studies including investigations of long-term effects of pressure waves. The brain injury was assessed by the biomarker S100b. Serum levels of S100b show a time-dependent increase following experimental blast traumatic brain injury.^9^ S100b was not elevated at any peak pressure, suggesting that the brain was not structurally damaged.

No macroscopic damage was found in lungs, intestines, or meninges. Detected microscopic hemorrhages were not correlated to the pressure wave. The hemorrhages were small and did not affect the cardiopulmonary function or circulation (pulse or oxygen saturation), and therefore not survival. Blast waves transiting material interfaces, especially in the transition to less dense materials, deposit energy near those interfaces. The majority of injuries occur in the lungs and the intestines through spalling of epithelium and microvasculature into air spaces. The high prevalence and severity of intestinal damage is unique to underwater blast injury.^12^ The organ injuries were less distinct than in previous experimental underwater studies. Richmond *et al*. reported 50% ruptured eardrums from 156 kPa-ms, 50% gastrointestinal hemorrhage from 172 to 186 kPa-ms and 50% pulmonary hemorrhage from 234 kPa-ms.^14^ It is possible that the isolated pressure wave from non-explosives differed to explosive-derived pressure waves. Likely, the large number of reflections in the model were not fully contributing to the damage level expected from this waveform. Most waveforms do not have this many reflections adding to the impulse. Future studies may further explore this aspect by include lining of the tube with non-reflective material such as Rho-C rubber. It is also possible that the position of the animals in relation to the pressure wave varied between the studies, which may be investigated in future studies by attaching pressure sensors to the animals.

The study has some limitations to be discussed. First, we did not investigate surface wave reflections. Most human injury was described near the surface of the water.^13^ A Friedlander equation that accurately describes a generalized underwater blast waveform and the surface rarefaction wave intersecting the primary waveform has not been defined and requires extensive investigations. This rarefaction wave can result in a decrease both in peak pressure and in overall impulse for measurement points near the surface of the water. Neither the exponential decay models nor the scaling laws can account for the negative pressure reflection off the surface.^13^ The aim of this study was to investigate the neurological impact from direct underwater pressure waves. Therefore, surface wave reflections were beyond the scope of this initial study. However, the water shock tube construction allowed for variations in distance to the surface, and surface wave reflections may be the focus of future studies. Second, we did not assess long term cognitive effects. The experimental setup allowed for the time under water to be extended, making the water shock tube well adapted for survival studies. Third, the animals were under general anesthesia, which may confound EEG, as discussed above. Fourth, the observation time was 30 min. The time span allowed for investigating immediate responses to pressure waves. However, we did not investigate delayed effects, and it is possible that an increase in S100b would occur after longer observation times. Fifth, the hood over the head of the rat did allow oxygen flow in front of the nose, which may distort the pressure wave in front of the nose. Sixth, we did not account for differences in mass. Experiments in animals need to be properly scaled by mass to relate them in a useful way to humans, and while the omission of mass scaling is the most common error in air blast experiments, it was not possible to perform the same scaling of the waveform duration in these experiments because the method for mass-scaling in underwater blasts is not yet known. This should be the focus of future studies.

In the current US Navy guideline, the given value for “probable risk of injury” is at a peak pressure higher than the pressure values reported from most of the fatalities evaluated in a comprehensive review by Lance *et al*.^13^ Given the stricter requirements necessary for protection against impaired consciousness and the ability to orient underwater, the effects on the brain may result in serious consequences and should therefore be considered when determining safety distances. The field of underwater blast injury needs solid, data-based guidelines that can be used by operators and medical personnel while in the field.^12^ The water shock tube construction produced a reproducible pulse, of sufficient amplitude and duration,^2^ which made the model well adapted for experimental injury assessments and did not require explosive substances. Injuries occur in water at long ranges compared with air blast, and long term sequelae from such events at much larger ranges than by air blast.^13,15^ It is possible that spatial- and temporal disorientation may cause death at pressure levels with low organ affection. A 50% risk of fatality from underwater blast at 302±16 kPa-ms impulse was derived from 262 human exposures, including a 20% risk of pulmonary injury at one kilometer from a 20 kg charge.^13^ We detected EEG changes at pressure levels by which mild organ hemorrhage occurred, warranting further investigations to determine neurological effects of underwater blast.

## References

[1] Andersen P, Loken S (1968). Lung damage and lethality by underwater detonations. Acta Physiol. Scand..

[2] Arborelius U., H. Almström, J. Bursell, A. Sondén and J. Persson. Development of an experimental model for study of the effects of under water shockwaves on the central nervous system and hearing organs. *FOI Swedish Defence Res. Agency* 1–24, 2004.

[3] Axelsson H, Hjelmqvist H, Medin A, Persson JK, Suneson A (2000). Physiological changes in pigs exposed to a blast wave from a detonating high-explosive charge. Mil. Med..

[4] Bass CR, Rafaels KA, Salzar RS (2008). Pulmonary injury risk assessment for short-duration blasts. J. Trauma.

[5] Boutillier J, Deck C, Magnan P, Naz P, Willinger R (2016). A critical literature review on primary blast thorax injury and their outcomes. J Trauma Acute Care Surg.

[6] Brown EN, Lydic R, Schiff ND (2010). General anesthesia, sleep, and coma. N. Engl. J. Med..

[7] Davidsson, J., U. Arborelius, L.G. Ohlsson, L. Kawa, N. Chye, J. Lu, and Risling. The clemedson blast tube. *Neuromethods* 149, 2019.

[8] Dewey JM, Sochet I (2018). The Friedlander Equations. Blast Effects: Physical Properties of Shock Waves.

[9] Gyorgy A, Ling G, Wingo D, Walker J, Tong L, Parks S, Januszkiewicz A, Baumann R, Agoston DV (2011). Time-dependent changes in serum biomarker levels after blast traumatic brain injury. J. Neurotrauma.

[10] Hayton SM, Kriss A, Muller DP (1999). Comparison of the effects of four anaesthetic agents on somatosensory evoked potentials in the rat. Lab. Anim..

[11] Inoue M, Ates N, Vossen JM, Coenen AM (1994). Effects of the neuroleptanalgesic fentanyl-fluanisone (Hypnorm) on spike-wave discharges in epileptic rats. Pharmacol. Biochem. Behav..

[12] Lance RM, Bass CR (2015). Underwater blast injury: a review of standards. Diving Hyperb. Med..

[13] Lance RM, Capehart B, Kadro O, Bass CR (2015). Human Injury Criteria for Underwater Blasts. PLoS ONE.

[14] Richmond DR, Yelverton JT, Fletcher ER (1973). Far-Field Underwater-Blast Injuries Produced by Small Charges.

[15] Ross JA, Macdiarmid JI, Osman LM, Watt SJ, Godden DJ, Lawson A (2007). Health status of professional divers and offshore oil industry workers. Occup. Med. (Lond.).

[16] Skotak M, Alay E, Chandra N (2018). On the accurate determination of shock wave time-pressure profile in the experimental models of blast-induced neurotrauma. Front. Neurol..

